# *Van*B-*Van*C1 *Enterococcus gallinarum*, Italy

**DOI:** 10.3201/eid1109.050282

**Published:** 2005-09

**Authors:** Caterina Mammina, Anna Maria Di Noto, Antonella Costa, Antonino Nastasi

**Affiliations:** * Università degli Studi, Palermo, Italy;; † Istituto Zooprofilattico Sperimentale della Sicilia, Palermo, Italy;; ‡ Università degli Studi, Florence, Italy

**Keywords:** vancomycin-resistant enterococci, poultry, vanB-vanC1, letter

**To the Editor:** We report detecting a *van*B determinant in *Enterococcus gallinarum* in poultry in Italy. High-level *van*A-mediated glycopeptide resistance has been described for *E. gallinarum* and *E. casseliflavus* (*1**–**4*), and *van*B-mediated vancomycin resistance has been frequently described for *E. faecalis* and *E. faecium*. However, *van*B-mediated resistance in isolates of *E. gallinarum* has been described only in sporadic nosocomial cases of infection or colonization (*5**,**6*).

In January 2005, a study of contamination by foodborne organisms in slaughtered broiler carcasses was conducted in Sicily. To detect glycopeptide-resistant enterococci (GRE), each carcass was placed in a bag with 100 mL sterile buffered peptone water and shaken vigorously for 60 sec. After overnight incubation at 37°C, 0.5 mL rinsate was added in duplicate to 5 mL ethyl violet azide broth (Oxoid, Basingstoke, United Kingdom) with 4 mg/L vancomycin. Broth cultures were further incubated at 37°C for 48 h, and 0.1 mL aliquots were spread onto duplicate plates of VRE (commercial denomination product, Oxoid) agar.

A vancomycin-resistant isolate of *E. gallinarum* was identified in a carcass from a broiler farm in eastern Sicily. The biochemical tests of API 20 Strep (bioMérieux, Marcy l'Etoile, France) and motility test at 30°C were used to characterize the isolate at the species level. The MICs of vancomycin and teicoplanin were 64 μg/mL and 1 μg/mL, respectively. The isolate was subjected to a multiplex polymerase chain reaction followed by an endonuclease cleavage of amplicons by *Msp*I (Invitrogen, Carlsbad, CA, USA) as previously described (*7*) to detect *van* gene determinants; this process demonstrated a simultaneous presence of *van*C1 and *van*B determinants.

*E. gallinarum* and the other motile enterococci are thought to infrequently cause infection. However, the recent involvement of *van*C1-*van*A *E. gallinarum* in person-to-person spread in a long-term-care facility (*8*) and in an intensive care unit (*2*), along with identification of *van*C1-*van*B isolates in some patients treated with prolonged courses of glycopeptides (*5**,**6*), suggests reassessment of their possible pathogenic role.

For the first time, 1 isolate of *E. gallinarum* has been found harboring the *van*B gene in poultry. Our findings confirm that *E. gallinarum* can capture the genetic determinants of high-level glycopeptide resistance, probably under selective pressure conditions that do not permit survival of a host organism with constitutive low-level resistance (*3*). Previous studies have demonstrated that *E. gallinarum* can transfer these determinants to *E. faecium* by conjugation (*2*).

The role of food animals as reservoirs of GRE and the causes of their persistently high prevalence in poultry carcasses in some European countries are being investigated (*9*). Moreover, the public health risk associated with consumer exposure to GRE when handling raw animal foods is poorly understood. In Europe, the food chain is thought to be the major source of GRE since avoparcin was used as a food additive for animals until the European Union ban in 1997. Previous studies in Italy showed that avoparcin withdrawal successfully reduced GRE contamination of poultry meat products (*10*). However, our finding, 7 years after the European Union ban, highlights that resistance genotypes in motile enterococci should be closely monitored (*11*).
